# Special Issue “State-of-the-Art Polymer Science and Technology in Japan (2021, 2022)”

**DOI:** 10.3390/polym15112576

**Published:** 2023-06-04

**Authors:** Shin-ichi Yusa, Naozumi Teramoto

**Affiliations:** 1 Department of Applied Chemistry, Graduate School of Engineering, University of Hyogo, 2167 Shosha, Himeji 671-2280, Hyogo, Japan; 2 Department of Applied Chemistry, Faculty of Engineering, Chiba Institute of Technology, 2-17-1 Tsudanuma, Narashino 275-0016, Chiba, Japan; teramoto.naozumi@it-chiba.ac.jp

It has been 100 years since the first article on polymerization was published by Hermann Staudinger. Polymer science contributes to the improvement of human life and culture, and polymer science and technology, which originated from the science and industrial technology of synthetic fibers in Japan, is now one of the most important research fields in the Japanese scientific community, with numerous Japanese research groups being active in various research fields related to polymer science and technology. Polymer science, both basic science and practical application, contributes to domestic and international economic development. Polymer technology, such as functional and high-performance polymer materials, covers a wide range of research fields such as electricity, electronics, information, biotechnology, medicine, transportation, architecture, and space applications. Furthermore, polymer science has recently been applied in various rapidly growing fields such as energy (e.g., fuel cells), the environment (e.g., marine plastic problems, carbon neutrality, futuristic materials’ development, and medical/healthcare), and IoT technology. Therefore, the development of polymer science and technology is expected to enable the creation of innovative science and technology, which will facilitate major changes in the world’s industry and society. We hope that this Special Issue will provide a representative concept for cutting-edge directions in polymer science and technology in Japan.

This Special Issue aims to provide an overview of research into polymer science in Japan. Both research papers and reviews illustrating recent achievements and advancements in polymer-related research recently performed in Japan are included. A brief account of the research presented in the issue is provided below.

Taguchi et al. [1] reported the ruthenium (II)-catalyzed polymerization of *t*-butyl 4-azido-5-hexynoate (*t*BuAH) to obtain copolymers composed of 1,5-substituted 1,2,3-triazole units (1,5-units) and 1,4-substituted 1,2,3-triazole units (1,4-units) with *M*_w_ ≈ 2.7–3.6 × 10^3^ oligomer. This is the first report on dense 1,2,3-triazole oligomers composed of 1,5-units linked via a carbon atom.

Rova et al. [2] investigated changes in the tensile properties of polybutylene succinate (PBS) and basalt fiber (BF)-reinforced PBS (PBS-BF) composite sheets during degradation in bacterial solutions. The PBS-BF composite specimens showed little change in ultimate tensile strength (UTS) after 56 days of immersion in the bacteria-free medium. In comparison, when immersed in the bacterial solution for 56 days, the UTS of the PBS-BF composite specimens decreased to about half of the UTS recorded immediately after preparation. 

Mizoue et al. [3] prepared amphiphilic diblock copolymers (PChM-PNIPAM) consisting of poly(cholesteryl 6-methacryloyloxyhexanoate) (PChM) and poly(*N*-isopropylacrylamide) (PNIPAM) blocks via controlled radical polymerization. The PChM and PNIPAM blocks showed liquid crystalline behavior and a low critical solution temperature (LCST), respectively. PChM-PNIPAM formed water-soluble polymer micelles in water below the LCST due to the hydrophobic interaction of the PChM block. 

Sato [4] reported on the lattice theory of block copolymer solutions near the boundary between the micellar and liquid–liquid phase-separation regions, which suggests a new kinetic process of micellization in which small, concentrated phase droplets are first formed in the initial stage of micellization and then transformed into micelles. 

For more than half a century, during the storage of celluloid paintings from animated films, the problem of paper sticking to acrylic paints has occurred. Kaneko et al. [5] established a methodology to cleanly remove paper from paint by evaluating the effect of solvents on peel behavior. 

Yano et al. [6] prepared chitin nanofiber (CHNF)-reinforced fish collagen peptide (FCP) hydrogels, which were prepared by photo-crosslinking methacrylic acid groups chemically bonded to FCP. The compressive and tensile properties of the FCP hydrogels were significantly improved; the tensile strength and modulus increased with increasing CHNF content. In cell culture assays, fibroblasts grew well on FCP hydrogels reinforced with CHNF. 

Kumakura et al. [7] reported that bio-based polyureas (PUs) with furan rings in the main chain were synthesized via the polyaddition reaction of 2,5-bis(aminomethyl)furan and various diisocyanates such as methylene diphenyl diisocyanate. The furan ring of the PU backbone underwent a dynamic Diels–Alder (DA) reaction with a bismaleimide (BMI) crosslinker. Rapid cooling from 80 °C to room temperature maintained the solution phase, and slow cooling reformed the gel. The recovered gel exhibited self-healing properties. 

Imoto et al. [8] reported that the oxidation of alcohols using 4-amino-TEMPO-immobilized monolith catalysts was investigated in batch- and continuous-flow reactions. Polymer monoliths were prepared via polymerization-induced phase separation using styrene derivatives, and 4-amino-TEMPO was immobilized on the polymer monoliths via a flow reaction. In flow oxidation, the eluent permeated without clogging, and efficient flow oxidation was possible with a residence time of 2–8 min. 

Stimuli-responsive polymeric nanoparticles (NPs) reversibly change their dispersion or aggregation state in response to external stimuli. Kataoka et al. [9] reported that core–shell NPs with a weakly charged shell containing threonine and a fluorescent crosslinked core were synthesized. Stable and homogeneous NPs (dTh) and NPs (Fl) consisting of a fluorescent symmetrical diphenyldithiophene (dTh) and diphenylfluorene (Fl) crosslinked core were prepared via a site-selective Suzuki coupling reaction. NPs with Fl in the core show high fluorescence intensity in different solvents, which can be regarded as aggregation-induced luminescent NPs, which show strong luminescence in the aggregated state of the crosslinked core. 

Nguyen et al. [10] investigated the self-assembly of pH-responsive random and block copolymers composed of 2-(*N*,*N*-diisopropylamino)ethyl methacrylate and 2-(methacryloyloxy)ethyl phosphorylcholine in aqueous media. In an acidic environment, these copolymers existed as a single polymer chain that did not interact with each other. In contrast, when the pH of the solution was raised above the critical value of 8, separated micelles were formed. 

Tabuchi et al. [11] reported that *N*-cyclohexylphthalimide-substituted trifluoroacetylamino (CF_3_CONH-) groups forming intramolecular hydrogen bonds were synthesized and exhibited bright yellow fluorescence by excited-state intramolecular proton transfer in solution and crystalline forms. CF_3_CONH-substituted phthalic anhydride was also synthesized and attached to the end of a semi-aromatic polyimide (PI) chain that exhibited blue fluorescence. This material design strategy is promising for the creation of thermally stable white fluorescent PIs that can be applied in solar cell spectral transducers, displays, and information and communication technology (ICT) devices. 

Watanabe et al. [12] reported the synthesis and absorption properties of homopolymers composed of 1,3,4,6,9b-pentaazaphenalene (5AP). Oxidative polymerization via the Scholl reaction was carried out, and homopolymers of various lengths were isolated. The absorption bands of homopolymers were obtained in a longer wavelength region than the monomer. The extensibility of the conjugated system via meta-substituted frameworks and the distance dependence of the main chain conjugation were evaluated. 

Finally, Tashiro et al. [13] reviewed the development of the crystal structure analysis of polymers via a hybrid method combining wide-angle X-ray diffraction (WAXD) and wide-angle neutron diffraction (WAND) with many case studies conducted by the authors. The development of the structural analysis of synthetic polymers using WAXD/WAND technology is described with a view to future perspectives.

This Special Issue presents recent research on polymer chemistry in Japan. A wide range of research is included in this Special Issue, such as the synthesis of new polymers, characterization, functional polymers, and so on. The 13 research papers in the Special Issue are not only written by Japanese authors. Several papers are products of international collaborations. It is firmly predicted that the Japanese polymer research field will continue to expand internationally.
